# Biofilm Lifestyle in Recurrent Urinary Tract Infections

**DOI:** 10.3390/life13010148

**Published:** 2023-01-04

**Authors:** Amr S. Abu Lila, Azza A. H. Rajab, Marwa H. Abdallah, Syed Mohd Danish Rizvi, Afrasim Moin, El-Sayed Khafagy, Shams Tabrez, Wael A. H. Hegazy

**Affiliations:** 1 Department of Pharmaceutics, College of Pharmacy, University of Ha’il, Ha’il 81442, Saudi Arabia; 2 Molecular Diagnostics and Personalized Therapeutics Unit, University of Ha’il, Ha’il 81442, Saudi Arabia; 3 Department of Pharmaceutics and Industrial Pharmacy, Faculty of Pharmacy, Zagazig University, Zagazig 44519, Egypt; 4 Department of Microbiology and Immunology, Faculty of Pharmacy, Zagazig University, Zagazig 44519, Egypt; 5 Department of Pharmaceutics, College of Pharmacy, Prince Sattam bin Abdulaziz University, Al-Kharj 11942, Saudi Arabia; 6 Department of Pharmaceutics and Industrial Pharmacy, Faculty of Pharmacy, Suez Canal University, Ismailia 41522, Egypt; 7 King Fahd Medical Research Center, King Abdulaziz University, Jeddah 21589, Saudi Arabia; 8 Department of Medical Laboratory Sciences, Faculty of Applied Medical Sciences, King Abdulaziz University, Jeddah 21589, Saudi Arabia; 9 Pharmacy Program, Department of Pharmaceutical Sciences, Oman College of Health Sciences, Muscat 113, Oman

**Keywords:** recurrent urinary tract infections, catheter-associated urinary tract infections, biofilm formation, biofilm eradication, anti-virulence agents

## Abstract

Urinary tract infections (UTIs) represent one of the most common infections that are frequently encountered in health care facilities. One of the main mechanisms used by bacteria that allows them to survive hostile environments is biofilm formation. Biofilms are closed bacterial communities that offer protection and safe hiding, allowing bacteria to evade host defenses and hide from the reach of antibiotics. Inside biofilm communities, bacteria show an increased rate of horizontal gene transfer and exchange of resistance and virulence genes. Additionally, bacterial communication within the biofilm allows them to orchestrate the expression of virulence genes, which further cements the infestation and increases the invasiveness of the infection. These facts stress the necessity of continuously updating our information and understanding of the etiology, pathogenesis, and eradication methods of this growing public health concern. This review seeks to understand the role of biofilm formation in recurrent urinary tact infections by outlining the mechanisms underlying biofilm formation in different uropathogens, in addition to shedding light on some biofilm eradication strategies.

## 1. Introduction

Urinary tract infections (UTIs) are one of the most common bacterial infections in humans, and account for 40% of all nosocomial infections [1,2,3,4]. The incidence of UTIs among the population has increased by 60% between the years 1990 and 2019, confirming that UTIs represent a common public health problem that is far from being eradicated [5]. Typical symptoms include bacteriuria accompanied by urinary frequency, urgency, dysuria and suprapubic discomfort [6]. UTIs can be classified into complicated and uncomplicated urinary tract infections. Uncomplicated UTIs, commonly called cystitis, are a lower urinary tract infection, which only involves the bladder. Such infections are simple and easily resolved with antibiotic treatments. On the other hand, a complicated urinary tract infection is a simple infection that has progressed to the upper urinary tract and is usually associated with other risk factors. Complicated UTIs usually require longer antibiotic courses and are associated with high rates of treatment failure, recurrent infection, sepsis, and significant morbidity and mortality [6,7,8]. Uncomplicated UTIs are common in females, affecting 40–60% of females at least once in their lifetime, while all urinary tract infections in males are considered complicated [6,8]. Recurrent UTIs are defined as at least two acute episodes of UTIs occurring within a 6-month period or three episodes in a 12-month period, with much higher incidence in females than males [9,10]. The infection can be community-acquired, hospital-acquired or self-infected [10,11,12]. Most community-acquired infections result from poor personal hygiene, low sanitary precautions, or multiple sexual partners. Self-infection usually arises in immunocompromised individuals or high-risk cases, usually caused by commensal inhabitants from the periurethral, vaginal or rectal flora [13,14].

Biofilm formation represents the cornerstone in the pathogenesis of UTIs, since bacterial biofilms play a pivotal role in catheter-associated UTIs (CAUTIs), which account for 40% of all nosocomial infections [15,16,17]. Furthermore, biofilm formation is considered one of the most important mechanisms behind the high rates of recurrence and antimicrobial resistance that are often associated with UTIs [15,17,18,19]. Bacterial pathogens, both Gram-negative and Gram-positive, are the most common suspects behind UTIs [9,19,20]. *Escherichia coli*, *Proteus mirabilis*, *Klebsiella pneumoniae*, *Staphylococcus epidermidis*, *Staphylococcus saprophyticus*, *Staphylococcus aureus* and *Enterococcus faecalis* are among the most frequent bacterial pathogens that are common in uncomplicated UTIs and complicated UTIs, as well as in CAUTIs [5,6,14,21,22,23]. This is not to mention the increasing role of *Pseudomonas aeruginosa* and *Acinetobacter baumannii* in complicated nosocomial UTIs [24,25]. However, some fungi, such as *Candida* spp., are also associated with high incidence of UTIs. Although using different pathogenic pathways, *Candida* spp. share bacterial pathogen with their ability to form persistent biofilms that contribute to chronic recurrent UTIs [11,26,27,28]. The current work aims to review UTIs’ pathogenesis, shedding light on the most frequent UTI pathogens, with special focus on biofilm lifestyle and its impact on pathogenesis of UTIs. Furthermore, the present review summons some of the more traditional approaches to eradicating biofilms in addition to the latest ones.

## 2. Urinary Tract Infections (UTIs)

### 2.1. Risk Factors

UTIs are very common infections, and one of the most common infections encountered in health care facilities. However, some risk factors are associated with high infection rates. The female gender represents an important risk factor; it is estimated that 40–60% of females will get a urinary tract infection at least once in their life, and half of them will experience recurrence within one year [8,9,10]. On the other hand, in males, the incidence of contracting UTI drops to 10–15%, and drops even further for circumcised males [6]. The risk of infection is higher in females due to many factors. First of all, females have a shorter urethra than males, which means a shorter distance that bacteria have to travel to reach the urinary bladder and initiate the infection [29]. Additionally, the incidence of self-infection from the perineal flora is higher in females, due to the anatomical differences between the two genders [8]. Hormonal fluctuations during menopause represent another female-related factor due to the drop in estrogen level, which makes urogenital skin thinner and reduces colonization by *Lactobacilli*. Colonization of *Lactobacilli* provides a protective barrier against pathogenic infection mediated by lactic acid production, which reduces vaginal pH, hence rendering the media less suitable for bacterial growth [30]. Pregnancy is another risk factor, due to reduced immunity [31]. Likewise, immunocompromised individuals and geriatrics are at particularly high risk [32,33]. Diabetes mellitus is one of the major risk factors contributing to high rates of complicated urinary tract infections, especially in catheterized cases [5,21]. This can be explained due to nephropathy, glucosuria and reduced immunity, which are common complications found in most diabetic patients [17]. Other risk factors include structural abnormalities of the urinary tract, renal stones, physical interventions like the use of catheters, spermicides, contraceptive diaphragms, intrauterine devices, and frequent pelvic examinations [6,14].

### 2.2. Microbial Etiology of UTIs

**Figure 1:**
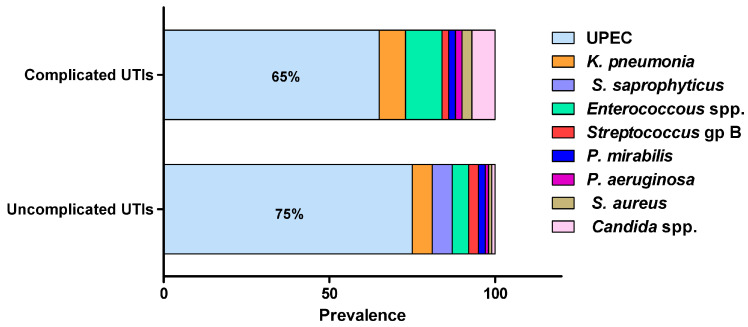
The most frequent uropathogens (adopted from Flores-Mireles, et al., 2015). UTIs are caused by a wide range of Gram-negative and Gram-positive bacterial pathogens as well as fungi. Uropathogenic *E. coli* (UPEC) is the most common causative agent for both uncomplicated (75%) and complicated (65%) UTIs. The most prevalent pathogens in uncomplicated UTIs, in order of prevalence, are *Klebsiella pneumoniae* (6%), *Staphylococcus saprophyticus* (6%), *Enterococcus faecalis* (5%), group B *Streptococcus* (3%), *Proteus mirabilis (2%)*, *Pseudomonas aeruginosa* (1%), *Staphylococcus aureus* (1%), and *Candida* spp. (1%). For complicated UTIs, the most prevalent pathogens are *Enterococcus* spp. (11%), *K. pneumoniae* (8%), *Candida* spp. (7%), *S. aureus* (3%), *P. mirabilis* (2%), *P. aeruginosa* (2%) and group B *Streptococcus* (2%).

Most urinary tract infections would result from self-infection; others are transferred from sexual partners, bad hygiene habits or hospital-acquired. UTIs can be caused by a wide range of Gram-negative and Gram-positive bacteria as well as fungi (Figure 1) [6,8,11,23,25]. The source of infection mostly arises from the normal commensal inhabitants of the urogenital, rectal and vaginal flora, with intestinal microbiota acting as the main reservoir of infection [8,23]. *E. coli* is by far the most commonly isolated uropathogen, accounting for 75% of uncomplicated UTIs and 65% of complicated UTIs [34]. *K. pneumonia* is the second-most common uropathogen, causing 6–8% of all UTIs, while *E. faecalis* and *C. albicans* are major causative agents of complicated UTIs, accounting for 11% and 7% of infections, respectively [14]. *Proteus mirabilis*, *Staphylococcus saprophyticus*, *Staphylococcus epidermidis*, *Staphylococcus aureus*, group B *Streptococcus*, *Pseudomonas aeruginosa* and *Acinetobacter baumannii* are less frequently isolated from UTIs [35].

### 2.3. Pathogenesis of UTIs

The infection starts by the ability of some bacteria to cross the urethral sphincter muscle, which acts as a natural barrier against pathogens, followed by adherence to the urethral epithelium [19]. Adhesion is mediated by bacterial fimbriae and adhesins, which play a pivotal role in the initiation of urinary tract infections. Later, most pathogens would climb up the urethra by swimming to the urinary bladder [14,22]. Colonization in the urinary bladder is attained by simultaneous expression of multiple virulence factors – mainly motility, adhesion and secretion of toxins like proteases, hemolysins, urease, colony-necrotizing factors, siderophores, and polysaccharide coatings. These toxins would reflect tissue necrosis and facilitate invasion, giving rise to a typical cystitis [23]. Common symptoms are bacteriuria accompanied by fever, dysuria, pyuria, itching, urinary frequency, urgency lower abdominal pain (LAP), suprapubic pain, burning sensation during urination, blisters and ulcers in the urogenital area [7,36].

### 2.4. Catheter-Associated Urinary Tract Infections (CAUTIs)

Urinary catheters are tubular devices made of latex or silicone that are widely used in hospitalized patients. Catheter-associated urinary tract infections (CAUTIs) are the most common nosocomial infections, accounting for approximately 40% of all hospital-acquired infections [5,21]. Catheters are foreign bodies that induce local mechanical stress which in turn activates a wide range of inflammatory responses at the adjacent tissues such as exfoliation, oedema, and mucosal lesions, in addition to localized deposition of serum fibrinogen on the catheter surface [37]. Fibrinogen acts as a lubricating layer with the aim of reducing abrasion, but also provides an ideal niche for the attachment of pathogens and as a nutrient source [22]. Urinary catheters can be contaminated during insertion due to inadequate application of aseptic techniques; additionally, long-term catheterization (more than seven days) represents a significant risk factor for development of urinary tract infections, since the surface of urinary catheters acts as an attractive niche for bacterial adhesion and proliferation [17].

## 3. Bacterial Biofilms

Bacterial biofilms are closed bacterial communities in which the bacteria are embedded in an extracellular polymer matrix secreted by bacteria itself. This viscous medium anchors the bacterial colony at the infection site and provides a protective shield against host defenses, as well as antimicrobial treatments [13]. Bacterial biofilm formation represents an important virulence factor in most uropathogens, playing an important role in infection persistence and recurrence [19]. Additionally, bacterial biofilms represent the main cause behind CAUTI, which is the most common complication in long-term hospitalized patients [38]. The bacterial population within the biofilm shows an altered behavior to that of planktonic bacteria; bacteria rearrange their priorities in order to fortify their attack plan, where motility and metabolic activities are reduced in order to conserve energy and nutrients [19,39]. At the same time, extracellular toxins are upregulated in order to reflect maximum possible tissue damage; this ensures generous release of nutrients at the infection site and further cements the biofilm in site [40,41]. Meanwhile, the biofilm community would continue to shed daughter planktonic cells that aim to seed adjacent tissues, causing the spread of infection and development of new biofilms [13]. Eventually, a bacterial biofilm would develop into a mature, densely-packed structure that is very difficult to eradicate, hence contributing to chronic and recurrent infections [19].

### 3.1. Biofilm Formation Mechanism

**Figure 2:**
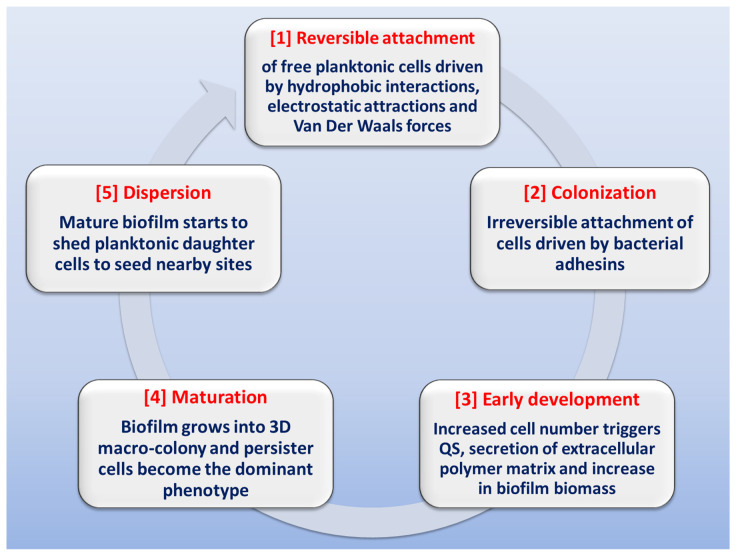
Schematic representation of the stages involved in biofilm formation.

The stages involved in biofilm formation have been extensively studied in order to identify possible mechanisms for biofilm inhibition (Figure 2) [13,28,40,41,42,43,44]. The first stage in biofilm formation is the reversible attachment of freely-swimming planktonic bacteria to a suitable surface, which can be found in a wide variety of sites or niches. Inanimate hydrophobic surfaces like polystyrene catheters, prosthetic devices, stents or rough surfaces like renal stones represent an ideal surface for biofilm attachment. This step is driven by different forces like hydrophobic interactions, Van der Waals forces, or electrostatic attraction. The attachment can be easily affected by media pH, temperature and solutes [42,45,46]. At some point, attachment becomes irreversible due to the stronger forces of adhesion mediated by bacterial adhesins. Noteworthy is the fact that inhibition of this step (by downregulation of pili formation or by adhesins antibodies) can drastically diminish biofilm formation [47]. The next stage is early development, in which the attached bacteria start replicating at site, causing an increase in population number, which triggers the initiation of QS communication systems; this cell-to-cell communication will result in increased secretion of extracellular polysaccharides. After this is the biofilm maturation stage, in which biofilm biomass grows into a three-dimensional macro-colony in which the dominant bacterial phenotype is the persister cells. At this stage, biofilm eradication is very difficult [48]. Finally, at the dispersion stage, the biofilm starts shedding daughter planktonic cells that seed adjacent sites for the formation of new biofilms [49,50].

### 3.2. Bacterial Communication within Biofilms

**Figure 3:**
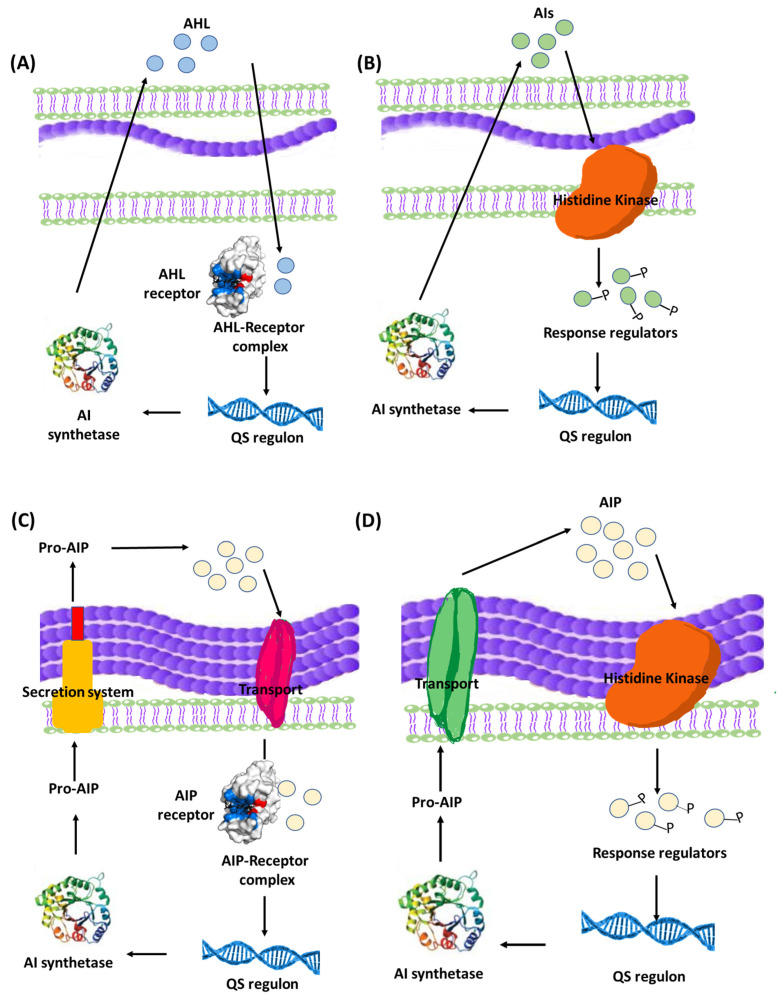
Bacterial QS systems in Gram-negative (**A**) two-component signaling, or (**B**) LuxI/LuxR-type QS systems. Gram-positive (**C**) two-component signaling, or (**D**) AIP-binding transcription factor QS systems. AI: Autoinducers, AIP: Autoinducing peptides.

Quorum sensing (QS) is a bacterial communication system which allows the bacteria to coordinate their behavior using chemical molecules as a communication signal. It is important to understand that QS communication system aims at orchestrating a grouped bacterial behavior which results in maximum benefits for the population within the biofilm including optimum use of nutrients, increased pathogenicity, and extension of survival rates [18,51]. That being said, it is logical to expect a certain threshold for activation of the QS system by reaching a minimum population level in order to trigger the onset of the system [41,45]. The mechanisms of QS and its role in controlling group bacterial behavior have been extensively studied, revealing that QS system utilizes an inducer/receptor mechanism to control bacterial gene expression [18,43,51,52,53]. The same system is used in both Gram-negative and Gram-positive bacteria. However, the signaling pathway is different. Gram-negative bacteria recruit a system based on N-acyl-homoserine lactones (AHLs) inducers that are synthesized via AHL synthase genes, such as different *luxI* genes, and sensed by their cognate receptors LuxR-type QS receptors [54,55]. The AHLs molecules are secreted in minute amounts that are undetected at low bacterial population. However, at high population levels, the amount of secreted AHLs is enough to bind to specific bacterial receptors, forming QS receptor-AHL complex. After hitting a certain threshold concentration, the QS receptor-AHL complexes control the transcription of genes involved in biofilm formation [56,57,58,59,60]. On a parallel basis, QS systems in Gram-positive bacteria employ autoinducing peptides (AIPs) that are detected by two-component membrane-bound signal transduction systems [55,61,62,63]. The different QS systems in Gram-negative and Gram-positive bacteria are represented in Figure 3. QS plays a crucial role in biofilm formation through controlling the production of extracellular polymer matrix, upregulation of bacterial virulence genes, increasing secretion of exoenzymes, siderophores, antibiotics and bioluminescence [50,64].

## 4. Biofilm Formation Patterns in Some Clinically Important Uropathogens

### 4.1. Escherichia coil

*E. coli* is the most common causative agent of both complicated and uncomplicated urinary tract infection in humans (accounting for around 65–75% of total cases), and one of the most common causes of Gram-negative bacteremia in hospitalized patients [14,19]. The colon represents a natural reservoir of multiple strains of *E. coli*, among which uropathogenic *E. coli* (UPEC), neonatal meningitis *E. coli* (NMEC), sepsis-associated *E. coli* (SEPEC), and avian pathogenic *E. coli* (APEC) can be found [34,65]. UPEC is characterized by multiple virulence factors which allow for adhesion and colonization of the urinary tract epithelium as well as tissue invasion. The initial adhesion of the pathogen to the uroepithelium is a crucial step for initiation of the infection. Adhesion is mediated by fimbrial adhesions, mainly type 1 fimbriae and *P. fimbriae* [47,66]. These adhesins also play an important role in cytokine induction, tissue inflammation and biofilm initiation [66]. Flagellar motility allows for climbing up the urethra, followed by colonization of the uroepithelium in the urinary bladder [66,67]. Hemolysin is secreted to induce tissue damage and siderophores are released to sequester iron from the damaged tissues [68]. The overall virulence behavior aims at localized tissue destruction and initiation of biofilm formation. Previous studies have confirmed the positive correlation between hemolytic activity, biofilm formation and higher levels of antimicrobial resistance in UPEC [69]. The infection triggers a strong response from the innate immunity resulting in cystitis, neutrophil influx and exfoliation of the superficial bladder epithelial as a defense mechanism [70,71,72]. 

In response, *E. coli* would take an alternative strategy by forming intracellular mini biofilm communities within immature epithelial cells, which can be detected by confocal microscopy as small clusters near the nucleus [70]. The intracellular pathway is initiated by bacterial FimH adhesin of type 1 pili, which recognizes uroplakins and integrins receptors on the superficial umbrella cells of the bladder epithelium, followed by activation of the RHO GTPases family and bacterial endocytosis [44]. Once inside the host cell, bacterial cells start to multiply, forming intracellular bacterial communities (IBC) whose formation is mainly responsible for recurrent infections and chronic cystitis [31]. Numerous studies also confirmed the role of K1 capsule polysaccharide of *E. coli* as an essential virulence factor for successful intracellular survival and IBC formation [31]. In later stages, after localized inflammation-triggered exfoliation of the infected layers, bacteria are released from the IBC by exocytosis and infect deeper exposed immature epithelial cells, hence formation of new IBC and persistence of the infection [44].

### 4.2. Klebsiella pneumoniae

*K. pneumoniae* are the second-most-common uropathogens after *E. coli*. The bacterium is known for its high adhesiveness and tendency to form biofilms on catheters and medical devices, making it a leading cause of nosocomial infections, mostly UTIs, pneumonia and septicemia, especially in immunocompromised individuals [73,74]. Biofilm formation in *K. pneumoniae* is strongly linked to a significant increase in multidrug resistance patterns, with many studies reporting a 10-to-1000-fold increase in the rate of multidrug resistance in mature bacterial biofilm as compared to planktonic cells [73,74]. The most characteristic virulence factors of *K. pneumoniae* are the thick capsular polysaccharide, in addition to type 1 and type 3 pili. The polysaccharide capsule in *K. pneumoniae* can reach a thickness 16 times that of the capsule of *E. coli* [75]. It consists of two fibrous layers: the inner one consists of thick, densely-packed fibers, and the outer layer, in which the fibers are less densely packed and become finer as they go outwards, forming a fine network on the capsule surface [74]. This thick fibrous capsule grants the bacterium multiple benefits: it forms a protective barrier surrounding the bacterial cell, provides the bacterium with extra protection against phagocytosis and serum complement deposition, reduces the penetration ability of antibiotics and bacteriophages, and boosts the adhesiveness of the bacterium onto mucus membranes and inanimate surfaces [74,76,77].

*K. pneumoniae* pili are involved in the initial adhesion to abiotic surfaces, which is followed by quick coverage of the surface caused by the entangled fibrous polysaccharide capsule of adjacent bacterial cells [78]. The combination of the high adhesiveness and high biofilm-forming ability of *K. pneumoniae*, in addition to its protective fibrous capsule, results in high resistance to disinfection, making this pathogen very challenging to eradicate in health care facilities—hence, the high incidence of nosocomial infection associated with this pathogen, especially in catheterized patients, which also results in high incidence of recurrent urinary tract infections and bacteremia in high-risk individuals [17,73]. 

### 4.3. Proteus mirabilis

*P. mirabilis* is a common uropathogen that accounts for 4–10% of all UTIs, and is considered the second-most-common causative agent of catheter-associated UTIs after *E. coli*, especially in long-term catheterized patients [5,21,22,79]. A characteristic feature of *P. mirabilis* is its unique ability to form crystalline biofilms that commonly lead to catheter encrustation and blockage and which, in most cases, is accompanied by urine retention and ascending UTIs [80]. In addition, *P. mirabilis* could express several virulence factors that predispose the occurrence of urinary tract infection. For instance, *P. mirabilis* can express multiple types of fimbriae and adhesins, including *P. mirabilis* fimbria (PMF), *P. mirabilis* p-like fimbria (PMP), mannose-resistant proteus-like (MR/P) fimbriae, mannose-resistant Klebsiella-like (MR/K) fimbriae, non-agglutinating fimbria (NAF), and ambient temperature fimbria (ATF) [67,81,82]. The aim of these multiple types is to be able to adhere to different types of surface materials in different media, giving rise to the bacterium’s remarkable stickiness to many surfaces and at different conditions [81]. 

The bacterium is known for its remarkable swarming motility, alternating between two main phenotypes: the swimmer and the swarmer phenotypes. The latter is characterized by heavy expression of flagella [79,83]. The importance of swarming motility in UTIs’ development remains controversial. However, it was reported that swarming cells were able to co-express other virulence factors, such as urease, ZapA protease, and hemolysin, which are all upregulated during swarming [67,84]. These enzymes aim at reflecting maximum tissue damage in order to facilitate invasion and the release of iron and other nutrients [83,85]. Needless to say, that motility plays an important role in ascending UTI and pyelonephritis that are common in *P. mirabilis* infections. The bacterium also shows remarkable ability to swarm across catheters made of silicone or latex, hence contributing to CAUTIs [79,82]. However, some studies reported that non-motile strains of *P. mirabilis* were able to induce UTI at similar rates to that of motile strains with comparable complications [85,86].

Urease secretion is another important virulence factor of *P. mirabilis*; the enzyme results in hydrolysis of urea and release of ammonia, which shifts the pH of urine into an alkaline environment, and subsequent precipitation of calcium and magnesium ions and the formation of urinary stones composed of magnesium ammonium phosphate (struvite) and calcium phosphate (apatite) [82]. The crystals provide a suitable surface for bacterial attachment and biofilm formation in which bacteria thrive and cause more crystal precipitation [79]. Eventually, the crystalline biofilm grows to the level of blocking urine flow through the catheter, and the formation of renal stones in the ureters, bladder and kidneys. The formed stones are directly related to higher rates of recurrent UTIs since they act as a perfect safe haven for the hiding of bacteria from host defenses and antimicrobial activity, as well as a suitable surface for attachment of other bacteria and formation of biofilms of multiple bacterial communities [79,83,87]. The stones would also block urine flow, causing urine retention, which further supports bacterial growth and multiplication (no flushing of urine) [79,83]. Additionally, some studies reported the ability of *P. mirabilis* to initiate an intracellular lifestyle within the urinary epithelium cells, accompanied by formation of intracellular calcium containing crystals; however, *P. mirabilis* fails to establish intracellular biofilms like those observed in *E. coli* UTI [87,88].

### 4.4. Enterococcus faecalis

*E. faecalis* is an opportunistic bacterium that is a common member of intestinal flora in humans and animals [14]. *E. faecalis* is a common causative agent of both community-acquired and hospital-acquired urinary tract infections (UTIs), with high rates of complicated UTI, especially in catheterized patients and patients under prolonged antibiotic treatment with vancomycin or third-generation cephalosporin [32]. The infection is likely to proceed into severe, life-threatening systemic infections in about 15–24% of cases, especially immunocompromised individuals [32]. Pathogenic *E. faecalis* usually originates from undercooked animal-based food, which is often linked to high rates of antimicrobial resistance due to the extensive use of antibiotics in animal farms [37,52]. The infection can also be transmitted from person to person by orofecal route [37]. *E. faecalis* is highly associated with CAUTIs, due to its significant ability to form biofilms on urinary catheters. The bacterium takes advantage of the fibrinogen deposition on the catheter surface and uses it as a nutrient source through the production of fibrinogen digestive proteases [89].

Some of the most important virulence factors in *E. faecalis* are the surface polysaccharide antigens that are essential for the initial attachment and subsequent biofilm formation on urinary catheters, mainly adhesin collagen of *Enterococcus faecalis* (Ace), enterococcal surface protein (Esp), and enterococcal biofilm associated pili (Ebp). The latter is of special importance, since it contains an N-terminal fibrinogen-binding domain which allows the bacterium to adhere firmly to the fibrinogen-coated catheter surface [37,89]. Another unique feature of *E. faecalis* is its ability to suppress the innate immune response at the site of infection. This is contrary to the strong inflammatory response that is expected upon insertion of a foreign body like a urinary catheter in the bladder, a response that is initiated by neutrophils and macrophages [32,37]. The immune modulating effect of *E. faecalis* is mediated by the simultaneous action of multiple virulence factors: (i) gelatinase enzyme, whose expression is turned on by QS, cleaves the complement components C3, C3a, and C5a, which inhibits opsonization and neutrophil recruitment, hence inhibiting pathogen labeling for subsequent phagocytosis; (ii) TcpF, a protein that interferes with Toll-like receptor (TLR) activity of leukocytes, hence inhibiting the subsequent initiation of both innate and adaptive immune pathways; and (iii) the aggregation substance (AS), which initiates internalization into macrophages and uroepithelial cells and confers resistance to super oxides, leading to increased intracellular survival within macrophages and bladder epithelium [32,37,52]. The overall result of the previous effects is the development of high-titer catheter-associated biofilms, as well as the ability to evade phagocytosis, decrease antibodies production, and survive within macrophages, neutrophils and bladder epithelium for extended periods [89]. Moreover, the immunosuppression at the infection site encourages co-infection with other uropathogens, hence promoting polymicrobial urinary tract infection [32]. This explains the general observation that *E. faecalis* is often found as part of a polymicrobial biofilm community and usually co-isolated with *E. coli* and other uropathogens like *P. aeruginosa* and *P. mirabilis*, leading to aggravated symptoms and higher risks of complicated UTIs [89]. *E. faecalis* has intrinsic resistance to trimethoprim, clindamycin, cephalosporins and penicillins, and lately has been associated with increased rates of resistance to glycopeptides, including vancomycin, which is considered a last-resort antibiotic against multidrug-resistant bacteria [14].

### 4.5. Staphylococcus *spp.*

*Staphylococci* are divided into two major groups: the coagulase-positive staphylococci which are mostly pathogenic, and the coagulase-negative staphylococci (e.g., *S. epidermidis* and *S. saprophyticus*), which are frequent inhabitants of the human gastrointestinal tract, cervix, urethra, vagina, perineum, and rectum [90,91]. One infamous member of the coagulase-positive staphylococci is *S. aureus*, which is an opportunistic inhabitant of the nasopharyngeal epithelium and axillae skin and flips easily to pathogenic form. Coagulase-negative staphylococci are normally less virulent and express fewer virulence factors. *Staphylococcus saprophyticus* is a common uropathogen associated with 10–20% of uncomplicated UTIs in sexually active young women [90]. This bacterium has high ability to survive in hostile toxic conditions, in addition to a remarkable tolerance to disinfection and heavy metal treatment. *S. aureus* represents one of the most common causes of infection at all body tissues. *S. aureus* has been associated lately with high levels of multidrug resistance, especially with the recent increase in prevalence of methicillin resistant *S. aureus* (MRSA) and vancomycin resistant *S. aureus* (VRSA), which are challenging to eradicate and usually associated with high mortality rates [92]. *S. aureus* has many virulence factors: (i) surface adhesins that allow for adhesion and biofilm initiation, (ii) surface structures that allows evading host immunity and escaping phagocytosis (capsular polysaccharide and immunoglobulin binding protein A); and (iii) toxins that induce tissue damage and immune evasion, e.g., leukocidin, α-toxin, β-toxin, enterotoxins and toxic shock syndrome toxin. *Staphylococci* are commonly associated with high incidence of biofilm formation that is mediated through the expression of a clumping factor which has high affinity for fibrinogen and fibrin proteins. This factor promotes attachment to blood clots, injured tissues, fibrinogen-coated catheters, and indwelling medical devices [91,92,93,94]. *Staphylococci* biofilms are problematic to eradicate with antibiotics alone, and often require removal of the device [92,93]. 

### 4.6. Candida albicans

*C. albicans* is a dimorphic fungus that is a common inhabitant of normal flora in skin and mucous membranes. It is also an opportunistic bacterium that takes advantage of immunosuppression to cause candidiasis in different tissues: the oral cavity, GIT, genitourinary tract, the ears, the bones, the eyes, wounds and blood [28,95]. Risk factors of urinary tract candidiasis increase with prolonged catheterization, age, female gender, intensive care admission, diabetes, prosthetic devices and implants, obstructive uropathy, neonates, and prolonged antibiotic treatment [27]. *C. albicans* is the leading cause of fungal urinary tract infections and accounts for up to 25% of all CAUTIs and 10–20% of all nosocomial infections with significant risk of development into a systemic infection with high mortality rates [11,27]. The pathogenicity of *C. albicans* is highly dependent on multiple virulence factors, namely polymorphism, adhesion to both biotic and abiotic surfaces, and biofilm formation [27]. Polymorphism refers to the ability of the fungus to switch between three morphological forms: (i) the yeast form (oval-shaped budding cells which is the normal commensal form and the infective form in virulent strains); (ii) hyphal form (an invasive filamentous form which harbors important adhesins for attachment to other cells, secretes large number of hydrolytic enzymes and plays a pivotal role in biofilm formation); and (iii) septate pseudohyphae (which is a transient form found within mature *candida* biofilms) [28,95]. The ability of *C. albicans* to initiate a pathogenic infection is highly dependent on switching to the filamentous form and on biofilm formation [28]. The initial attachment of the fungus is mediated by two groups of adhesins: the agglutinin–like sequences adhesins (AlS) and the Hypha-associated GPI–linked protein adhesins (Hwp1) [26,27]. After adhesion, the yeast cells begin a stage of proliferation and filamentation accompanied by secretion of extracellular matrix material, the filamentous hyphal cells adhering to each other, forming support scaffolds that contribute to the overall mechanical stability of the biofilm [26,28,95]. 

Moreover, the biofilm matrix material contains the polysaccharides mannan and glucan. The latter plays a critical role inhibiting neutrophil activation and creates a thick physical barrier to prevent the diffusion of drugs. Furthermore, glucan has been proven to bind to amphotericin B, thus restricting its effectiveness [26,28]. *Candida* biofilms are associated with high levels of extracellular enzymes secretion, including lipases, esterases, and phospholipases hemolysins, all of which contribute to pathogenicity by inducing host tissue damage, releasing nutrients and increasing tissue invasion [11,27,28]. In addition, *C. albicans* produces a group of secreted aspartyl proteases (Saps) secreted by the filamentous morphotype, which causes tissue damage allowing for elongation of the filaments and tissue penetration to deeper layers, as well as degrading antibodies and blocking complement activation [28]. Furthermore, *C. albicans* biofilms are inherently resistant to most known antifungal drugs and show much-reduced sensitivity to amphotericin B, making biofilm-mediated candidiasis difficult to eradicate with high recurrence rates [26,95].

## 5. Contribution of Biofilm Formation to Pathogenicity in Urinary Tract Infections

Bacterial biofilm formation represents an important virulence factor in uropathogens, playing an important role in infection persistence and recurrence [19]. Bacterial biofilms represent the main cause behind catheter-associated urinary tract infection, which is the most common complication in long-term hospitalized patients [18,39,42]. During a biofilm infection, simultaneous activation of both innate and acquired host immune responses may occur. While the free planktonic bacterial cells can be eradicated by the host immune response or antibiotics or a combination of both, the bacterial population within the biofilm remains highly protected and continues to reflect serious host tissue damages [16,18]. There are multiple mechanisms that make biofilms a hazardous complication that contributes to the pathogenicity and persistence of the infection. Some of these mechanisms are briefly discussed in the following points.

### 5.1. The Extracellular Polymer Matrix

Consisting mainly of extracellular secreted polysaccharides (e.g., cellulose, polyglucosamine and alginates) in addition to a wide variety of proteins, glycoproteins, and glycolipids, extracellular DNA enzymes, signaling compounds, adhesins, nutrients, cell debris, wastes and surfactants [42,96]. The polymer matrix is highly hydrated, providing a viscous mucoid consistency that offers high adhesiveness to solid surfaces in addition to increased bacterial aggregation [19,97]. The overall result is increased biofilm biomass, increased viscosity, stronger attachment and bacterial proliferation within the biofilm [45]. The high viscosity of the biofilm prevents penetration of host immune defenses as well as antimicrobial treatments, thus contributing to the high resistance and persistence of biofilms [42]. 

### 5.2. Extracellular Toxins

Bacterial behavior within the biofilm is under quorum sensing control, resulting in upregulation of virulence factors that further fortify pathogenicity, so extracellular toxins are upregulated in order to reflect maximum possible tissue damage; this ensures generous release of nutrients at the infection site and further cements the biofilm in site [40,41].

### 5.3. Persister Cells

The limited availability of nutrients and oxygen within the biofilm results in sub-optimal bacterial growth rate and asynchronous microbial growth, giving rise to variant bacterial phenotypes within the biofilm. The most abundant phenotype is the persister cells that are known for their inherent resistance to antibiotics [18,38,39,96,98,99,100,101]. Persister cells are dormant, slow-growing cells with reduced metabolic activity and high tolerance to antibiotic treatment; these cells allow for fair distribution of nutrients, since their requirements are minimal. Additionally, they show altered metabolic pathways that result in loss of target site of most antibiotic treatments [48]. Moreover, the phenotypic variations among the biofilm-forming bacteria results in differential gene expression, giving rise to a cocktail of bacterial responses including toxin release, efflux systems activation, ion sequestering, and lipid biosynthesis, in addition to antibiotic resistance [45,50]. These different responses would serve as a rich pool for selection of phenotypes with the highest survival rates; eventually, natural selection would favor the most virulent and resistant bacteria that will dominate the bacterial population within the biofilm [41,50,102,103].

### 5.4. Horizontal Gene Transfer

**Figure 4:**
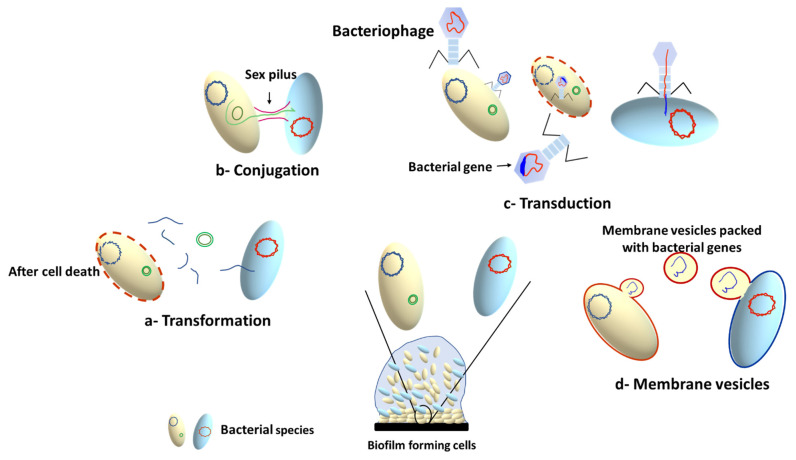
Horizontal gene transfer mechanisms in microbial biofilms.

The high bacterial density within the biofilm results in increased rate of horizontal gene transfer (HGT) between members of the biofilm community, which can take place between cells from the same or different species. This involves exchange of genetic elements such as transposons [104], plasmids [105], bacteriophages [106] and integrative conjugative elements (ICEs) [107]. There are three main mechanisms for HGT, depending on the type of transferred DNA: conjugation (for transfer of conjugative plasmids and ICE), transformation (for transfer of short pieces of chromosomal DNA and non-conjugative plasmids), and transduction (for bacteriophage mediated gene transfer) [106,108,109]. In addition to the three HGT mechanisms, membrane vesicles (MVs) have been recently documented as a cargo carrier that can mediate interspecies HGT, changing the expression of biofilm-related genes, and transferring antibiotic resistance genes [110,111]. The MVs can transfer DNA or sRNA that can encode diverse genes involved in virulence, stress response, and antibiotic resistance [111]. A schematic summary of the main mechanisms involved in HGT is presented in Figure 4. 

### 5.5. Resistance of Biofilms to Antibiotics

The resilient resistance of biofilms to antibiotics is a serious complication that is commonly implicated in most recurrent UTIs. Biofilms show an increased resistance to antibiotics that is estimated to be 10 to 1000 times greater than that of planktonic cells [112]. The resistant nature of biofilms is attributed to the collective effects of multiple factors: (i) the extracellular polymer matrix which confers a physical protective barrier against antibiotic penetration, hence diminishing the ability of antibiotics to reach hidden bacteria within the biofilm [18,39,96,113]; (ii) secretion of antibiotic inactivating enzymes that are upregulated under quorum sensing control [98]; (iii) phenotypic variations within the biofilm and the formation of persister cells which are inherently resistant to antibiotics as a result of their suppressed metabolic activity; (iv) increased expression of efflux pumps, which are membrane-bound transport proteins involved in the expelling of toxic molecules outside of the bacterial cell, including antimicrobial molecules; and (v) horizontal gene transfer (HGT) of antibiotic resistance genes. The HGT examples and their role in antimicrobial resistance development in biofilms are widely reviewed in many previous studies [108,109,110,114,115]. Additionally, efflux pumps allow the microorganisms to organize their internal environment by removing wastes, metabolites and quorum sensing signal molecules [116]. Furthermore, the biofilm bacteria share virulence factors’ encoding genes that make them reveal physiological and morphological changes [18,26,105,117]. For instance, the depletion of nutrients and oxygen in the biofilms leads to sub-optimal bacterial growth rates and shift in the phenotypic variations towards the formation of the persister cells. Interestingly, biofilms may be formed as a defensive consequence to the presence of antibiotics; for instance, the persistence of antibiotics as aminoglycosides, tetracycline, or cephradine at sub-inhibitory concentrations induce *E. coli* and *P. aeruginosa* to form biofilms [113,118].

## 6. Strategies for Eradication of Biofilms

Biofilm formation is a serious complication in most UTIs, and their eradication cannot be achieved by traditional antibiotic treatment, especially in case of aggressive or combined infections [18,73,96]. In addition, the worldwide increase in the rate of microbial resistance to antibiotics has turned into a global crisis that mandates the search for new strategies aimed at inhibiting and/or diminishing biofilm formation. In the next section, some biofilm eradication strategies will be discussed. 

### 6.1. Targeting QS System

QS system plays a pivotal role in controlling diverse bacterial virulence factors. In particular, it orchestrates the formation of microbial biofilms at different stages of infection [119,120]. Additionally, QS regulates the production of different exoenzymes and other virulence factors in biofilms [40,53,121]. That is why targeting QS is a promising approach to diminishing biofilm formation, as well as minimizing bacterial virulence, which would pave the way for the immune system to eradicate the infection [41]. Targeting QS system guarantees attenuation of the bacterial virulence that could ease the immunity task in eradication of bacterial infections and their biofilms [122,123]. Using anti-QS agents at sub-inhibitory concentrations is crucial for the success of the strategy, since higher concentrations would affect bacterial growth, which is considered a stress factor that directs bacteria towards developing resistance to the used antibiotics [58,122,123,124]. As described in an earlier section, the QS system works in an inducer-receptor manner (Figure 3), and its blockade can be achieved by: (i) blocking of biosynthesis of inducers, (ii) inactivation of the inducers, and (iii) interference with involved QS receptors and involved proteins [50,125,126,127]. In this direction, several chemical moieties and natural products were screened for their ability to block QS receptors and downregulate the expression of autoinducers [128,129,130,131,132,133,134,135,136,137]. 

The amino donor S-adenosyl methionine (SAM) is used in the production of homoserine lactone ring moiety (AHLs) in different Gram-negative bacteria [138,139,140]. SAM analogs (such as S-adenosylhomocysteine, S-adenosylcysteine, and other AHL analogs) can act as effective competitive inhibitors of the biosynthesis of AHL molecules, thereby diminishing bacterial virulence and biofilm formation [139,141]. On a similar basis, macrolide antibiotics such as azithromycin and erythromycin showed a significant ability to inhibit the biosynthesis of AHLs in *P. aeruginosa*, with subsequent diminishing of biofilm formation when used at sub-minimum inhibitory concentrations [142,143]. Likewise, it was reported in multiple studies that some chemical moieties showed a considerable ability to block QS receptors through downregulation of the autoinducer synthetase encoding genes. One example is observed in gliptins—a widely-used group of anti-diabetic agents—which were screened for their anti-QS activities. Notably, sitagliptin was shown to significantly downregulate the genes encoding autoinducer synthetase in *S. aureus*, *P. aeruginosa* and *Serratia marcescens*, which resulted in blocking of QS receptors and subsequently diminished biofilm formation and virulence expression [62,144,145,146]. Another approach that involved enzymatic degradation of QS inducers by AHL lactonases was also studied; such enzymes were able to degrade the homoserine lactone ring and stop QS signaling system, which in turn reflected a significant inhibition of bacterial virulence and biofilm inhibition [147,148,149]. 

Another technique involves the downregulation of the genes encoding QS receptor/inducer system encoding genes, which would eventually diminish bacterial virulence as well as biofilm formation [51,56,150,151]. It was shown that in *Pseudomonas aeruginosa*, downregulation of the Lux-type QS components LasI/R and RhlI/R, and non-Lux-type QS PqsA/R, confers significant inhibition of virulence as well as biofilm formation [62,130,137,145,152,153]. *Salmonella* and *E. coli* rely on Lux-type homologous SdiA to sense diverse AHLs; interestingly, mutations in the *sdiA* gene lead to significant reduction in the production of biofilms [46,136,154]. Similarly, ATP synthetase is an essential compartment in the Agr QS system in *Staphylococcus* spp., and its targeting leads to significant inhibition of biofilm formation [62].

### 6.2. Enzymatic Degradation of Biofilms

Using enzymes for biodegradation of biofilm polymer matrix is an attractive strategy to counteract mature biofilms. The biofilm matrix is formed basically from polysaccharides, proteins and DNA; this DNA is necessary for the initial attachment and aggregation of biofilm matrix and required for biofilm formation and regulation [155,156]. DNase is a degrading enzyme that can break down the phosphodiester linkage of DNA embedded in biofilm matrix resulting in effective biofilm [157]. Indeed, the antibiofilm activity of the DNase enzyme was repeatedly studied and showed significant biofilm inhibition against both Gram-negative and Gram-positive bacteria [158]. As another example, alginate lyase is an enzyme obtained from a marine bacterium that has a potent antibiofilm activity against *P. aeruginosa* and *E. coli* [159,160]. Additionally, DNase and alginate lyase effectively disintegrate the biofilm of *E. faecalis* and *E. faecium* [161]. Other examples include glycosidases and proteases which are degrading enzymes that are produced by bacterial cells themselves. Such enzymes can be used for hydrolytic degradation of biofilm matrix material and, hence, dismantling the enclosed bacterial community of their protective polymer coat. Some proteases such as proteinase K, serratiopeptidase, carboxypeptidase, α-amylase and others have also been screened for their anti-biofilm activities and showed significant biofilm eradication ability [162,163,164]. 

### 6.3. Downregulation of Biofilm Formation Pathways

As described previously, QS represents the master coordinator of all bacterial virulence behavior, especially biofilm formation. The ability to control transcription of genes involved in biosynthesis of biofilm components is of upmost importance for suppression of biofilm formation. Considering this approach, the interruption or downregulation of essential proteins that are involved in biofilm matrix secretion could lead to weaker biofilm consistency. Another example is observed in the ATP-binding cassette (ABC) transporters which are required for the initial steps of biofilm formation in *Pseudomonas aeruginosa* [165]. Targeting of this system by the synthetic antimicrobial peptide (Nal-P-113) results in decreased biofilm formation [166]. On a similar basis, the downregulation of genes responsible for anaerobic biofilm formation (e.g., *nirS*, *flgB*, *norC*, and *nosZ*), fucose binding of lectin (*lecB*), and secretion system Type IV pilus (*fimX*) would reflect biofilm inhibition in different bacterial spp. [167,168,169]. Other factors that are essential for biofilm initiation, such as motility and adhesion, represent a logical target when aiming for biofilm inhibition, based on the fact that non-motile bacterial cells or pili-deficient mutants cannot form biofilms [84,170,171]. Hence, downregulation of the genes that encode flagellar or fimbrial proteins leads to crippling of bacteria and suppression of biofilm formation. Several agents were tested in this regard. Sitagliptin and prazosin showed the ability to downregulate the genes *fimA*, *fimC* and *bsmB* that encode fimbria biosynthesis, and genes *flhD, rssB and rsmA* that control *S. marcescens* swarming. As a side effect, biofilm formation was significantly hindered as well [144,145,153].

### 6.4. Physical and Mechanical Methods for Eradication of Biofilms

The initial steps involved in biofilm formation are highly dependent on finding a suitable niche for bacterial attachment and subsequent biofilm establishment. Naturally, the surface characteristics play a decisive role in biofilm establishment; thus, manipulation of surface characteristics represents a possible approach for inhibition of biofilm formation, with the advantage of avoiding the use of biocides for cleaning and disinfection, hence decreasing the risk of bacterial resistance development [172]. The catheter surface is one of the most favorable niches for attachment of biofilms, thus physical modifications of the surfaces of the catheters could prevent bacterial adhesion and subsequent biofilm formation. This can be achieved by modification of physical or chemical properties of biomaterials creating anti-adhesive catheters [172]. Some studies reported the application of bio-inspired techniques to provide slippery liquid-infused porous surfaces (SLIPS), which have a super-hydrophilic nature that completely inhibits bacterial attachment. Similarly, the Sharklet topography technique can be applied to modify the surface of catheters and other biomedical devices [173]. Antifouling compounds such as hydrophilic polymers, betaine-based zwitterionic polymers and amphiphilic polymers have showed considerable anti-adhesive effects. Such agents can be used for coating catheter surfaces in order to hinder bacterial colonization and build-up of biofilms.

As for already-established biofilms, other physical interventions can be employed. An innovative photodynamic approach was tested for the ability to disintegrate mature biofilms. This approach assumes that the implementation of photosensitizing molecules to absorb the light intensity of specific wavelengths leads to targeting of bacterial cellular components [174,175]. These photosensitizing molecules produce reactive oxygen radicals that oxidize bacterial components and eventually reflect lethal effects on the bacterial community within the biofilm [175,176]. Electrochemical treatment is another technique that achieved promising results regarding biofilm matrix disintegration. The application of a weak electric field in the presence of antibiotics resulted in the enhanced penetration of antibiotics into the biofilm and eradication of the enclosed bacterial community using lower antibiotic concentration, in addition to biofilm destabilization and its eventual detachment as a result of the electrostatic forces generated under the direct current [177,178]. In addition, the electric current would induce hydrolysis and ionization of the biofilm media, which has a lethal effect on microbial cells via electrophoresis and electro-osmosis [179]. The electrochemical treatment technique was successfully employed for the eradication of *Acinetobacter baumannii* mature biofilms that are established on porcine explant [180]. The same technique was also successfully used for the eradication of *P. aeruginosa* biofilms formed on glass-bottomed petri dishes [181]. Nonetheless, despite these promising results, further investigations are required in order to allow for the simplification of such techniques, to facilitate their application in health care facilities in a realistic manner.

### 6.5. Bacteriophages

Phage therapy is one of the promising approaches that can be applied for eradication of aggressive multidrug-resistant infections [182]. Phage therapy has many advantages over traditional antibiotic therapy: (i) multiplication at the site of infection, which means that a very low dose will multiply and increase at the infection site with minimum concentration at other tissues [182,183,184]; (ii) viral tropism leads to high specificity in selection of the target bacteria species and even specific strains within the same species [184,185,186]; (iii) bacteriophage tropism ensures safety, since bacteriophages cannot infect eukaryotic cells; (iv) phage cocktails can be used to allow for a broader spectrum of activity; and (iv) high effectiveness against multidrug-resistant bacteria because bacteriophages use different pathways than antibiotics so they are not affected by bacterial resistance mechanisms [187]. However, owing to the lack of institutional authorizations for use of phage therapy on humans, their use is currently limited to food industry and agriculture applications. Another rewarding application is the inhibition of bacterial biofilms formed on inanimate surfaces [188]. The penetration of bacteriophages through biofilm matrix can be explained due to their minute size, which allows them to cross through water channels within biofilms and replicate eradicating bacterial cells deep within the biofilm. Additionally, some phages will direct bacterial cells to release depolymerizing enzymes that degrade the polysaccharides matrix of biofilms [189]. Furthermore, bacteriophages can infect persister cells, preventing their relapse reactivation [184].

However, some studies reported that bacteriophages are more effective in preventing biofilms rather than eradications of a well-performed one [190,191]. This is put down to low penetrability of bacteriophages into older biofilm layers [192], which may be due to slow bacterial proliferation and growth resulting in decreased bacteriophage efficiency [187,193]. In compliance, one study reported that early application of bacteriophages resulted in significant inhibition of *P. mirabilis* biofilm formation for over eight days and prevented the blockade of the used urinary catheters [194], while the late application of bacteriophages onto well-developed biofilms did not cause significant eradication of the biofilm [87]. Bacteriophages are ubiquitous in nature and in the digestive tracts of humans and animals. Numerous studies have reported the isolation of many bacteriophages showing anti-biofilm activities, with some examples listed in Table 1.

### 6.6. Drug Repurposing

Drug repurposing is a new strategy that has attracted the interest of researchers. This approach involves the screening of already-used and -approved medications for therapeutic applications other than their original indications. This approach offers numerous advantages, including: (i) discarding the need for clinical trials and authorization from health organizations since these agents are already approved and tested for their safety; (ii) decreasing the cost of treatment because the same medication can be used for multiple therapeutic indications; and (iii) identification of active chemical groups which allows for synthesis of chemical derivatives with reduced side effects [195]. Drug repurposing has attained a lot of attention in our research group over the years, where we were able to publish numerous promising results on the subject. Some of the screened agents showed noticeable antimicrobial activities which, when used at sub-inhibitory concentrations, revealed significant reduction of QS and biofilm formation. Some of the first medicinal drugs screened in our team were the group of α- and β-adrenergic blockers that were originally used for treatment of cardiovascular diseases [131,132]. However, after screening for their antimicrobial effects, the results revealed potent anti-QS and anti-biofilm activities. Drugs such as terazosin, doxazosin, prazosin, atenolol, and timolol showed significant reduction of the biofilms formed by *E. coli*, *P. mirabilis*, *P. aureginosa*, *Salmonella enterica*, and *S. marcescens* [131,132,136,153]. Gliptins were another group of drugs originally used as antidiabetic agents—they act as dipeptidase inhibitor (DPI-4). Sitagliptin is a member of this group that showed significant inhibition of *S. aureus*, *P. aureginosa* and *S. marcescens* biofilms [62,144,145,146,196]. Notably, both adrenergic blockers and gliptins inhibited biofilm formation by targeting the QS system and downregulation of its encoding genes. Furthering this avenue of inquiry, different chemical moieties of drugs were screened for their anti-biofilm activities [129,197,198]. Other drug groups were also screened, such as anti-inflammatory agents, mucolytics and diuretics, with many members showing interesting results. Some of these results are presented in Table 1.

### 6.7. Natural Products

Plants represent a rich source of bioactive compounds that are known as phytochemicals. Phytochemicals gained an increasing interest as anti-biofilm agents due to their abilities to quench QS, diminishing the adhesion and virulence of biofilm-forming bacteria [199]. Broadly, there are a few main classes that acquire anti-biofilm properties: phenolics, terpenoids, essential oils, alkaloids, lectins, polypeptides, and polyacetylenes [200]. However, some phytochemicals showed considerably decreased effectiveness and resistance development, which is a common outcome of their excessive widespread use [201]. They are employed efficiently as adjuvants to traditional antibiotics to eradicate microbial biofilms [202]. Garlic oil is one of the most well-known phytochemicals, which interferes with *Pseudomonas* and *Vibrio* spp. QS systems and biofilms [203]. Sotolon is a lactone with an extremely powerful aroma extracted from fenugreek seeds, which blocked the QS by downregulating its encoding genes [130]. Several examples of phytochemicals that showed biofilm inhibition activities are listed in Table 1.

### 6.8. Antimicrobial Peptides (AMPs) and Antimicrobial Lipids (AMLs)

Antimicrobial peptides (AMPs) are small proteins that play a defensive role as a member of innate immune response in many organisms. AMPs were suggested as promising anti-biofilm agents because of their ability to disrupt cell membranes by inducing membrane depolarization and perforation, which increases membrane permeability, enabling better penetration of antimicrobials and biofilm inhibitory agents [204,205,206]. The structure and shape of AMPs are critical to assure electrostatic interactions to interrupt cell membrane and biofilm [207]. Antimicrobial Lipids (AMLs) are known as single-chain lipid amphiphiles, including fatty acids and monoglycerides [208]. The AMLs also manipulate membrane permeability via destruction of the electron transport chain and by targeting the bacterial signal transduction systems, leading to loss of membrane selectivity, increased perforation and eventually, cell lysis [208,209]. The antimicrobial applications of AMPs and AMLs have been repeatedly examined against several bacteria [210,211,212].

An intracellular signal molecule “alarmone” is produced in stressed bacteria in response to drastic conditions. In harsh conditions, stringent factors convert uncharged tRNA to alarmones. The archetypical alarmone 5′-diphosphate 3′-diphosphate guanosine (ppGpp) which binds to RNA polymerase changes the preference of promoter, and hence changes the transcription rates [213,214,215]. It was documented that the stringent response could regulate the biofilm formation in *E. coli* and *S. mutans*, suggesting that degrading alarmone signal could lead to inhibition of biofilm formation [215,216,217,218]. Other peptides, like the synthetic cationic peptide LL-37, and D-enantiomeric peptides DJK-5 and DJK-6, were also reported for their anti-biofilm activities [169,219].

### 6.9. Nanoparticles

The technology of nanoparticles has gained much attention recently, with different applications in various fields. The minute size of the nanoscale offers the ability for high penetration capacity through various barriers, in addition to the ability to apply ultra-thin coats to specific target surfaces. The nanoscale size can modify the properties that ensure high capacity for targeting nanoparticles [220]. Cationic dendrimeric peptides’ surfaces are positively charged, that can be electrostatically adsorbed on the negatively charged surface of bacterial cells [168,221]. The hyperbranched nature of dendrimers enhances their chemical reactivity with the interacting surfaces, conferring therapeutic properties [168,222]. In addition to small particle size and high penetrating power in biofilm matrix, dendrimers can target DNA [223], and inhibit protein synthesis, exerting an efficient anti-biofilm activity [224,225]. The combination of nanoparticle technology and anti-biofilm agents (such as repurposed drugs, phytochemicals and anti-befouling agents) offers combined advantages from both methods, which increases penetration and the targeted selectivity of the used method. Some examples of the employed nanoparticles as anti-biofilms are listed in Table 1.

**Table 1:** Examples of antibiofilm agents.

Agent	Target Microbe	Expected Mode of Anti-Biofilm Action	Reference
**Repurposed drugs**			
**α-Adrenoreceptor blockers**			
Terazosin	*P. aeruginosa* *S. enterica*	Antagonize QS system	[131] [136]
Prazosin	*P. aeruginosa, P. mirabilis,* *S. marcescens, Salmonella enterica*	Antagonize QS system	[153,226,227]
β-Adrenoreceptor blockers			
Metoprolol Atenolol	*P. aeruginosa, S. enterica* *P. aeruginosa, P. mirabilis, S. marcescens*	Antagonize QS system	[132,228]
Antidiabetics			
Sitagliptin	*P. aeruginosa* *S. aureus* *S. marcescens*	Antagonize QS system	[62,144,229] [62] [146]
Saxagliptin	*S. mutans*	Enzymatical quenching	[230]
Metformin	*P. aeruginosa* *S. marcescens*	Antagonize QS system	[145] [146]
Diclofenac (analgesic)	*P. mirabilis*	Antagonize QS system	[135]
**Antipsychotics**			
Fluoxetine Thioridazine Penfluridol	*P. mirabilis* *P. mirabilis* *E. faecalis*	Interfering with biofilm formation molecular system	[231,232] [233]
**Anti-amoebic**			
Metronidazole Secnidazole	*P. mirabilis* *S. marcescens*	Antagonize QS system	[234] [235]
Allopurinol (anti-gout)	*P. aeruginosa*	Antagonize QS system	[152]
Ambroxol (mycolytic)	*S. marcescens*	Antagonize QS system	[128]
**Antibiotics**			
Azithromycin	*P. aeruginosa*	Antagonize QS system	[124]
Ciprofloxacin	*S. enterica*	Antagonize QS system	[236]
Resveratrol (anticancer)	*S. aureus* *E. coli* *Vibrio cholera*	Downregulation of biofilm formation molecular system	[237] [238] [239]
Ribavirin (antiviral)	*C. albicans*	Interfering with biofilm formation molecular system	[240]
Theophylline (bronchodilator)	*C. albicans*	Enzymatical quenching	[241]
**Phytochemicals**			
Stolon (fenugreek)	*P. aeruginosa*	Antagonize QS system	[130]
Garlic extract	*P. aeruginosa*	Antagonize QS system	[203]
Allicin (Garlic)	*P. mirabilis*	Antagonize QS system	[242]
Carvacrol (Oregano)	*P. aeruginosa*	Antagonize QS system	[243]
Emodin (*Polygonum cuspidatum*)	*S. aureus* *C. albicans*	Downregulation of biofilm formation molecular system	[244] [245]
Phytol (piper)	*S. marcescens*	Antagonize QS system	[246]
Curcumin (curcuma)	*Acinetobacter baumannii, C. albican, P. mirabilis*	Downregulation of biofilm formation molecular system	[247]
Tannic acid	*E. coli*	Disruption of biofilm matrix	[248]
Xylitol	*S. marcescens*	Antagonize QS system	[249]
Sodium citrate	*P. aeruginosa*	Antagonize QS system	[137]
Isolimonic acid (citrus fruits)	*E. coli*	Downregulation of biofilm formation molecular system	[250]
Zingerone (ginger)	*P. aeruginosa*	Antagonize QS system	[251]
Vanillin	*Chromobacterium violaceum*	Antagonize QS system	[252]
**Bacteriophages**			
vB_SauM_ME18 and vB_SauM_ME126	*S. aureus*	-	[199]
*Pseudomonas* Phage	*P. aeruginosa*	-	[183]
pSp-J and pSp-S	*Staphylococcal* spp.	-	[253]
Bacteriophage K	*S. aureus*	-	[184]
Bacteriophage cocktail	*P. aeruginosa*	-	[254]
Bacteriophage cocktail	*P. mirabilis*	-	[194]
vB_PmiS-TH	*P. mirabilis*	-	[185]
vB_EcoS-Golestan	*E. coli*	-	[186]
PhiS1	*P. aeruginosa*	-	[187]
PhiE2005-A	*P. aeruginosa*	-	[191]
Lytic bacteriophages cocktail	*P. mirabilis, E. coli*	-	[190]
Lytic bacteriophage	*K. pneumonia*	-	[192]
Staphylococcus bacteriophage K	*S. epidermidis*	-	[193]
**Antimicrobial Lipids (AMLs)**			
Glycerol monolaurate	*Haemophilus influenzae, S. aureus*	Disruption of biofilm matrix	[255]
Alginate oligosaccharide	*P. aeruginosa*		[256]
**Antimicrobial Peptides (AMPs)**			
AMP 1018	*S. mutants* *E. coli*	Disruption of biofilm cell membranes	[216] [217]
LL-37	*E. coli*		[207]
DJK-5	*P. aeruginosa*		[219]
DJK-6	*P. aeruginosa*		[219]
**Nanoparticles (NPs)**			
Ciprofloxacin-mesoporous silica	*S. enterica*	-	[236]
Metformin-silver NPs	*S. aureus*	-	[257]
Thymol nano-emulsion	*Salmonella* spp.	-	[134]
Gold nanoparticles (GNPs)	*E. coli, S. aureus, K. pnumonia*	-	[258]
SeNPs	*P. mirabilis*	-	[259]
ZnO:MgO NPs	*P. mirabilis*		[260]
Amphotericin B-PEG-ZnO	*C. albicans*	-	[220]
Nigella sativa-Zn NPs	*E. coli*	Antagonize QS system	[261]
Punica granatum -AgNPs	*E. coli, P. aeruginosa, P. mirabilis, S. aureus*	-	[262]
